# An Fgr kinase inhibitor attenuates sepsis-associated encephalopathy by ameliorating mitochondrial dysfunction, oxidative stress, and neuroinflammation via the SIRT1/PGC-1α signaling pathway

**DOI:** 10.1186/s12967-023-04345-7

**Published:** 2023-07-20

**Authors:** Yuqiang Liu, Han Yang, Nanbo Luo, Yifei Fu, Fang Qiu, Zhenglong Pan, Xiongjuan Li, Wenling Jian, Xinping Yang, Qingsheng Xue, Yan Luo, Buwei Yu, Zhiheng Liu

**Affiliations:** 1 Department of Anesthesiology, Shenzhen Second People’s Hospital/The First Affiliated Hospital of Shenzhen University, Shenzhen, China; 2 Department of Burn and Plastic Surgery, Shenzhen Longhua District Central Hospital, Affiliated Central Hospital of Shenzhen Longhua District, Guangdong Medical University, Shenzhen, Guangdong China; 3 Department of Anesthesiology, Ruijin Hospital Affiliated to Shanghai Jiaotong University, Shanghai, China

**Keywords:** Fgr, Sirtuin 1, PGC-1α, Mitochondria, Sepsis-associated encephalopathy

## Abstract

**Background:**

Sepsis-associated encephalopathy (SAE) is characterized by diffuse brain dysfunction, long-term cognitive impairment, and increased morbidity and mortality. The current treatment for SAE is mainly symptomatic; the lack of specific treatment options and a poor understanding of the underlying mechanism of disease are responsible for poor patient outcomes. Fgr is a member of the Src family of tyrosine kinases and is involved in the innate immune response, hematologic cancer, diet-induced obesity, and hemorrhage-induced thalamic pain. This study investigated the protection provided by an Fgr kinase inhibitor in SAE and the underlying mechanism(s) of action.

**Methods:**

A cecal ligation and puncture (CLP)-induced mouse sepsis model was established. Mice were treated with or without an Fgr inhibitor and a PGC-1α inhibitor/activator. An open field test, a novel object recognition test, and an elevated plus maze were used to assess neurobehavioral changes in the mice. Western blotting and immunofluorescence were used to measure protein expression, and mRNA levels were measured using quantitative PCR (qPCR). An enzyme-linked immunosorbent assay was performed to quantify inflammatory cytokines. Mitochondrial membrane potential and morphology were measured by JC-1, electron microscopy, and the MitoTracker Deep Red probe. Oxidative stress and mitochondrial dysfunction were analyzed. In addition, the regulatory effect of Fgr on sirtuin 1 (SIRT1) was assessed.

**Results:**

CLP-induced sepsis increased the expression of Fgr in the hippocampal neurons. Pharmacological inhibition of Fgr attenuated CLP-induced neuroinflammation, the survival rate, cognitive and emotional dysfunction, oxidative stress, and mitochondrial dysfunction. Moreover, Fgr interacted with SIRT1 and reduced its activity and expression. In addition, activation of SIRT1/PGC-1α promoted the protective effects of the Fgr inhibitor on CLP-induced brain dysfunction, while inactivation of SIRT1/PGC-1α counteracted the benefits of the Fgr inhibitor.

**Conclusions:**

To our knowledge, this is the first report of Fgr kinase inhibition markedly ameliorating SAE through activation of the SIRT1/PGC-1α pathway, and this may be a promising therapeutic target for SAE.

**Graphical Abstract:**

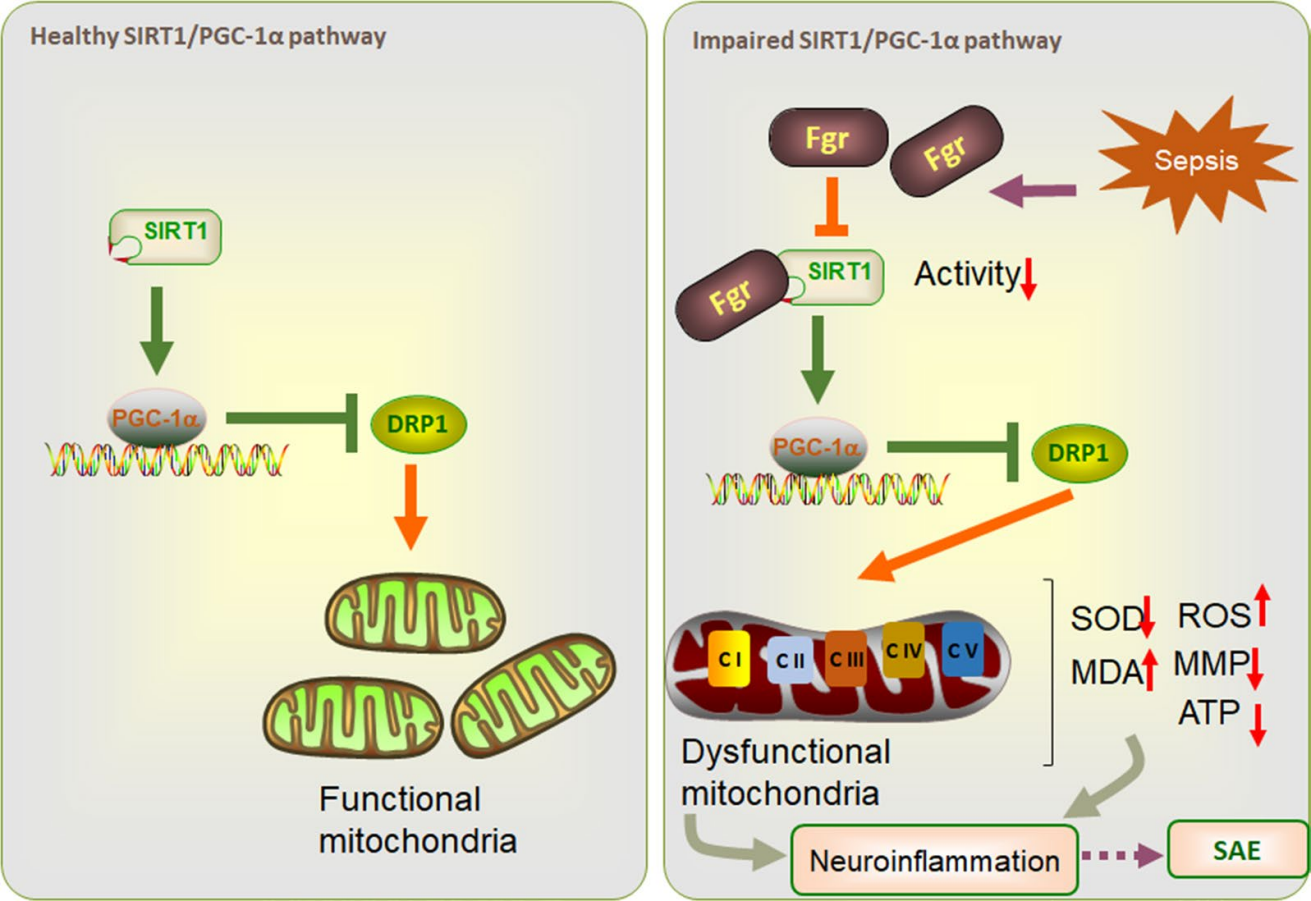

## Background

Sepsis is a life-threatening response to infection [1]. There are at least 19 million cases of sepsis worldwide every year, and half of these patients will never recover. Among the patients that never recover, 6 million die and approximately 3 million have cognitive impairment [2, 3]. Sepsis-associated encephalopathy (SAE) is a diffuse brain dysfunction secondary to sepsis without central nervous system infection. SAE occurs in approximately 70% of patients in intensive care units (ICUs) and it is associated with increased morbidity and mortality [4]. The clinical manifestations of SAE are delirium, cognitive impairment, anxiety, depression, and other behaviors. The hospitalization cost of sepsis survivors is as high as 98 thousand yuan [5], and SAE brings a substantial economic and mental burden to patients, their families, and the wider society. Management of SAE predominantly relies on good care in the ICU because there is no specific treatment for the disease. Neuron mitochondrial damage and neuroinflammation overactivation were reported to be associated with SAE [6]; however, the pathological mechanism(s) has not been fully elucidated. Thus, understanding the underlying mechanisms of SAE may reveal new perspectives for the therapeutic management of this disease.

Mitochondria are essential organelles for various biological processes, including energy production, metabolite biosynthesis, cell death, and immune responses [7]. Mitochondrial dysfunction is involved in cell death and the development of various human diseases. Damage to the electron transport chain is an essential factor in the pathogenesis of many neurodegenerative diseases [8]. Mitochondrial dysfunction and mitochondrial ultrastructural damage have been found in septic immune cells, cardiomyocytes, skeletal muscle, and hepatocytes [9]. In addition, ATP depletion and inhibition of the intracellular antioxidant system and respiratory chain (electron transport chain) have been reported in patients with sepsis and in animal models of the disease [10, 11]. Persistent mitochondrial dysfunction is associated with sepsis-related organ failure and poor prognosis in patients with sepsis [12]. Thus, restoring mitochondrial function may improve organ function in sepsis.

Fgr, which is a member of the Src family of tyrosine kinases, is highly expressed in many cancers and can regulate mitochondrial oxidative phosphorylation [13, 14]. Recent evidence has shown that inhibition of Fgr kinase with TL02-59 potently suppresses acute myelogenous leukemia (AML) cell growth in vitro and in vivo [15]. Pharmacological inhibition or genetic knockdown of thalamic Fgr attenuated hemorrhage-induced thalamic pain through NF-κB- and ERK1/2-triggered activation of microglia [16]. However, the role of Fgr in neuroinflammation and mitochondrial function has not been assessed in SAE. Consequently, this study aimed to investigate the potential role of Fgr in SAE.

## Methods and material

### Animals

Male C57BL/6 mice (8- to 10-weeks-old) were obtained from Guangdong Provincial Laboratory Animal Centre (Guangdong, China). All mice were housed in a standard room with a 12:12 dark/light cycle at 23 ± 1 °C. All experiments were approved by the Committee on the Animal Research Ethics of Shenzhen Second People’s Hospital (Guangdong, China).

### Cecal ligation and puncture (CLP) model

Mice were subjected to CLP according to a previously described method [17]. Briefly, mice were anaesthetized with pentobarbital intraperitoneally (i.p.) before surgery. After laparotomy, the caecum was ligated with a 4.0 suture and punctured with a 21 G needle. A small amount of feces was gently squeezed out of the cecum, and the cecum was placed into the abdominal cavity and sutured. Mice were resuscitated by injecting prewarmed normal saline (5 mL/100 g) subcutaneously immediately after surgery. Sham mice were subjected to the same procedure except for ligation and puncture. After 7 days, behavioral tests were conducted with the mice.

### Cell culture

A mouse hippocampal neuronal cell line (HT22 cells) was obtained from the Sun Yat-Sen University. The cells were incubated with Dulbecco’s Modified Eagle Medium (DMEM) medium containing 10% fetal bovine serum (FBS) (GIBCO BRL, Rockville, MD, USA) and 1% antibiotics (penicillin/streptomycin, 100 U/mL; GIBCO, Waltham, MA, USA).

### Drug administration

TL02-59 (an Fgr inhibitor), (R) EX-527 (a SIRT1 inhibitor), SRT 1720 (a SIRT1 activator), ZLN005 (a PGC-1α activator), and SR-18292 (a PGC-1α inhibitor) were obtained from MedChemExpress (Monmouth Junction, NJ, USA). Lipopolysaccharide (LPS) was purchased from Sigma (St. Louis, MO, USA).

TL02-59 was dissolved in 90% saline, 5% solution HS-15, and 5% *N*-methyl-2-pyrrolidone. TL02-59 (at 1 mg/kg, 10 mg/kg, or 15 mg/kg) and vehicle were administrated once daily for 3 days via the tail vein at 1 h after CLP. The CLP mice were injected i.p. with ZLN005 or SR-18292 at 12 mg/kg or 45 mg/kg after TL02-59 treatment. In the HT22 cells, TL02-59 (1 μM) was administered to cells for 24 h in the presence or absence of LPS (1 μg/mL). To determine whether (R) EX-527 or SRT 1720 treatment could reverse the protective effects of TL02-59, HT22 cells were incubated with (R) EX-527 (10 μM) or SRT 1720 (1 μM) for 24 h in the presence of LPS and TL02-59. To determine whether ZLN005 or SR 18292 treatment could reverse the protective effects of TL02-59, HT22 cells were incubated with ZLN005 (2 μM) or SR 18292 (2.5 μM) for 24 h in the presence of LPS and TL02-59.

### Open field test (OFT)

Mice were placed into the central area of a 50 × 50 × 50 cm white polyvinyl chloride box and allowed to move freely for 10 min. The total area was divided into 16 equal squares (4 × 4). The arena was recorded and analyzed using EthoVision XT automated tracking software (Nodus Information Technology, Wageningen, Netherlands).

### Novel object recognition test (NORT)

Mice were habituated into a 50 × 50 × 50 cm box for 10 min on the first day and 5 min on the second day. On the third day, the mice were placed in the box with two identical objects and allowed to freely explore for 5 min (sample phase). Next, the apparatus and objects between mice were cleaned. After 2 h retention, the mice were placed into the same box for exploration for 5 min (acquisition phase), during which one of the objects was replaced by a novel object. The number of mouse explorations of objects in the two phases was recorded. The recognition index was calculated as the percentage of counts spent exploring the novel object over the total exploration counts during the acquisition phase.

### Elevated plus maze (EPM)

A plus maze with a height of 50 cm consisted of two open arms (35 × 6 cm) and two closed arms (35 × 6 cm) with a 15 cm wall and a square central area (6 × 6 cm). Mice were placed into the central platform facing a closed arm and were allowed to explore for 5 min. The maze was cleaned with 75% ethanol after each trial. The performance of each mouse was recorded and analyzed using EthoVision XT automated tracking software. The number of arm entrances and time spent in each arm were recorded.

### Immunofluorescence staining

For staining in brain slices, mice were anaesthetized with sodium pentobarbital and perfused transcardially with phosphate-buffered saline (PBS), followed by 4% formaldehyde. Brains were post-fixed with 4% formaldehyde overnight at 4 °C, then placed in 30% sucrose solution. Sections were obtained on a microtome (Leica, Wetzlar, Germany) at 30 μm. Subsequently, the sections were washed with PBS, blocked (3% donkey serum and 0.2% Triton X-100 in PBS), and incubated overnight at 4 °C with primary antibodies diluted in blocking solution, washed with PBS, followed by incubation with secondary antibodies conjugated with Alexa Flour 555 and 488, respectively. Immunofluorescence images were captured by confocal microscopy (LSM 800, Carl Zeiss, Oberkochen, Germany) and analyzed using Image J software. The following primary antibodies were used: Fgr (1:50, Cat# DF3081, RRID:AB_2835464, Affinity Biosciences, Changzhou, China), SIRT1 (1:200, Cat# 8469S, RRID:AB_10999470, Cell Signaling Technology, Beverly, United States), PGC-1α (1:200, Cat# sc-518025, RRID:AB_2890187, Santa Cruz, Dallas, TX, USA), DRP1 (1:200, Cat# 8570S, RRID:AB_10950498, Cell Signaling Technology, Beverly, United States), NeuN (1:500, Cat# MAB377, RRID:AB_2298772, Millipore, Darmstadt, Germany), NeuN (1:500, Cat# ab177487, RRID:AB_2532109, Abcam, Cambridge, United Kingdom), GFAP (1:500, Cat# 3670, RRID:AB_561049, Cell Signaling Technology, Beverly, United States), and Iba1 (1:500, Cat# ab5076, RRID:AB_2224402, Abcam, Cambridge, United Kingdom).

### Western blotting

Mouse brain tissues or HT22 cells were homogenized in RIPA buffer (Beyotime, Shanghai, China) containing protease and phosphatase inhibitors (Roche, Mannheim, Germany) on ice and stored at − 80 °C until use. Protein concentrations were determined using a BCA assay kit (Thermo Fisher, Waltham, MA, USA). The proteins were separated via sodium dodecyl sulfate-polyacrylamide gel electrophoresis (SDS-PAGE) and transferred to a polyvinylidene fluoride (PVDF) membrane (Millipore, Darmstadt, Germany). After blocking, the membranes were incubated with primary antibodies at 4 °C overnight, then were washed with TBST and incubated with appropriate secondary antibodies (anti-Rabbit: 1:3000, Cat# 7074S, RRID:AB_2099233, Cell Signaling Technology; anti-Mouse: 1:3000, Cat# 7076S, RRID:AB_330924, Cell Signaling Technology). Proteins were visualized using a chemiluminescent reagent (Amersham-GE, Pittsburgh, USA). The following primary antibodies were used (dilution, source): Fgr (1:500, Cat# DF3081, RRID:AB_2835464, Affinity Biosciences), SIRT1 (1:1000, Cat# 8469S, RRID:AB_10999470, Cell Signaling Technology), PGC-1α (1:1000, Cat# 66369-1-Ig, RRID:AB_2828002, Proteintech, Wuhan, China), DRP1 (1:1000, Cat# 8570S, RRID:AB_10950498, Cell Signaling Technology).

### Quantitative RT-PCR

Total RNA from mice hippocampi was isolated using TRIzol regent (Invitrogen, Carlsbad, CA, USA) and was reverse transcribed into cDNA using ReverTra^®^ Ace qPCR RT Master Mix with gDNA Remove kit (TOYOBO, Osaka, Japan). The RT-qPCR was performed using SYBR^®^ Green Real-time PCR Master Mix (TOYOBO, Osaka, Japan) and data were analyzed using the 2^−ΔΔCt^ method. The primer sequences were as follows: Src (forward: 5′-TGCGGCTGGAGGTCAA-3′; reverse: 5′-TGCCTGGCTTCAGAGTTTT-3′), Lyn (forward: 5′-AGATCCAACGTCCAATAAACAG-3′; reverse: 5′-GGGTATAAGGCCACCACAAT-3′), Lck (forward: 5′-GTCCGCCATTACACCAACG-3′; reverse: 5′-GCCGCTCCACCAACTTCA-3′), Fyn (forward: 5′-ACGGACGGAAGATGACCTG-3′; reverse: 5′-CGTAATTGCTGGGAATGTAAC-3′), Hck (forward: 5′-GATTGCTGACTTTGGACTGG-3′; reverse: 5′-CACTTGATGGGGAACTTGG-3′), Yes (forward: 5′-AGGCTGCTCTGTATGGTCG-3′; reverse: 5′-CATTCTGTATCCCCGCTCTA-3′), Fgr (forward: 5′-CTCAAGGCCGGACTTCGTC-3′; reverse: 5′-CCCACCAGTCATACTCCGTATTG-3′), and β-actin (forward: 5′-TTTGCAGCTCCTTCGTTGC-3′; reverse: 5′-CCATTCCCACCATCACACC-3′).

### Enzyme-linked immunosorbent assay (ELISA)

The mice hippocampal tissues were homogenized in cold PBS and the supernatants were collected and stored at − 80 °C until processing. The levels of interleukin (IL)-6 and tumor necrosis factor (TNF)-α in the brain were analyzed using mouse ELISA kits (SAB, MD, USA) according to the manufacturer’s instructions.

### Transmission electron microscopy (TEM)

Hippocampal tissues were fixed with 2.5% glutaraldehyde for 24 h at 4 °C. After washing with PBS, the tissues were fixed with 1% OsO_4_ for 2 h at room temperature, then dehydrated in ethanol, embedded in Epon-Araldite resin for penetration and placed in a model for polymerization. After the semi-thin section was used for positioning, an ultrathin section was made and collected for microstructure analysis. Followed by the counterstaining of 3% uranyl acetate and 2.7% lead citrate. Sections were observed with an HT7800 transmission electron microscope (Hitachi, Ltd., Tokyo, Japan).

### Mitochondrial morphology observation

HT22 cells were seeded in 6-well plates. After treatment with SIRT1 or PGC-1α activator/inhibitor, the cells were incubated with MitoTracker Deep Red (500 nM, Cat# 8778S, Cell Signaling Technology) for 30 min at 37 °C. Images were captured by confocal microscopy (LSM 800, Carl Zeiss).

### Mitochondrial membrane potential

HT22 cells were seeded in 6-well plates and treated with SIRT1 or PGC-1α activator/inhibitor. Next, the cells were incubated with JC-1 dye (200×, Beyotime, Shanghai, China) for 20 min at 37 °C. After washing, images were captured by confocal microscopy (LSM 800). JC-1 monomer fluorescence was detected at 529 nm with an excitation wavelength of 514 nm. The fluorescence of JC-1 aggregates was detected at 590 nm with an excitation wavelength of 585 nm. The fluorescence intensity ratio of JC-1 aggregate/JC-1 monomer was calculated to detect changes in mitochondrial membrane potential. Images were analyzed using Image J software.

### Detection of reactive oxygen species (ROS) production in vivo and in vitro

Dihydroethidium (DHE) (UElandy, Shuzhou, China) was used to detect the production of ROS in the hippocampus. Frozen sections were washed with PBS and incubated with 5 μM DHE for 1 h in the dark. Subsequently, the sections were washed and mounted with ProLong Gold Antifade Mountant (Thermo Fisher) and the red fluorescence was examined by confocal microscopy.

Intracellular ROS levels were detected by a ROS assay kit (Beyotium) according to the manufacturer’s instructions. Briefly, after treatment with SIRT1 or PGC-1α activator/inhibitor, HT22 cells were incubated with DCFH-DA for 20 min at 37 °C and washed with DMEM medium. The cells were then observed by confocal microscopy, with green fluorescence detected at 488 nm. Images were analyzed using Image J software.

### Detection of superoxide dismutase (SOD) activity and malondialdehyde (MDA) levels

HT22 cells were collected and homogenized. The protein concentrations in the homogenates were determined using a BCA assay kit (Thermo Fisher). Then, the SOD activity and MDA levels were measured by using SOD and MDA assay kits (Beyotime), respectively, according to the manufacturer’s instructions.

### ATP measurement

Hippocampal tissues or HT22 cells were homogenized and ATP levels were detected using an ATP assay kit (Beyotime) according to the manufacturer’s instructions.

### Co-immunoprecipitation

Hippocampal tissues were obtained, lysed, centrifuged, and the supernatants were collected. Co-immunoprecipitation was performed using a Dynabeads Protein G IP kit (Thermo Fisher) according to the manufacturer’s instructions; 3 μg of anti-Fgr (Santa Cruz) and anti-SIRT1 (Abcam) were used. Western blotting was subsequently performed as described above.

### Mitochondrial complex I–V activity assay

Mice hippocampal mitochondria were isolated and assayed using the CheKine™ Micro Mitochondrial Complex Activity Assay Kit (Abbkine, Wuhan, China) according to the manufacturer’s instructions.

### Kinase activity assay

Fgr kinase activity was measured using a Universal Kinase Activity Kit (R&D Systems, USA) according to the manufacturer’s protocol. Recombinant mouse Fgr protein and different truncated SIRT1 proteins were obtained from Origene company. Shp2 protein was obtained from Sino Biological. Briefly, the proteins were added into the final reaction for 20 min at room temperature, then the optical density was measured at 620 nm using a VICTOR Nivo Multimode Microplate Reader (PerkinElmer). A positive control (ADP instead of ATP), a negative control (assay buffer instead of kinase), and a blank (no substrate and kinase) were included in the assay.

### SIRT1 activity assay

Nuclear extracts from hippocampal tissues and HT22 cells were obtained using a nuclear extraction kit (Epigentek, Farmingdale, NY, USA) according to the manufacturer’s instructions. Subsequently, SIRT1 activity in the nuclear extracts was measured using an Epigenase Universal SIRT Activity/Inhibition Assay Kit (Epigentek).

### Data analysis

Data were expressed as the mean ± SEM and were analyzed using GraphPad Prism 7 software (GraphPad Software, La Jolla, CA, USA). Statistical significance was estimated using an unpaired Student’s *t*-test and one-way analysis of variance (ANOVA). A *p*-value < 0.05 was considered to be statistically significant.

## Results

### Mouse hippocampal Fgr expression is increased after sepsis induction

**Fig. 1:**
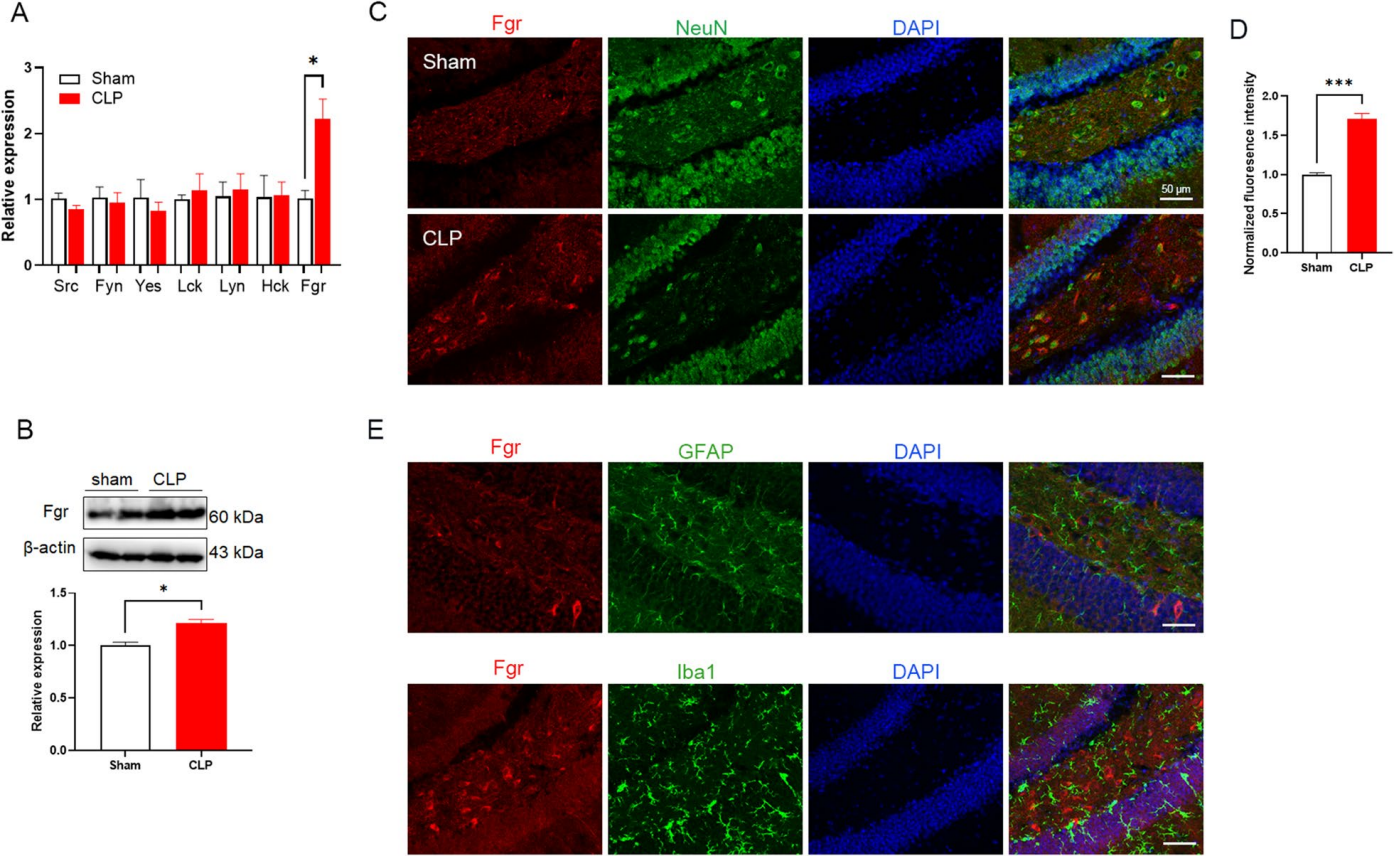
Hippocampal Fgr was increased in the CLP mouse model. **A** The mRNA levels of Src family kinase members in the hippocampus after CLP surgery (*n* = 3 for control mice; *n* = 4 for CLP mice; mean ± SEM, unpaired *t*-test, *p < 0.05). **B** Level of Fgr protein in the hippocampus of septic mice (*n* = 3, mean ± SEM, unpaired *t*-test, *p < 0.05). **C**, **D** Representative fluorescent images (**C**) and quantitative analysis (**D**) of Fgr in hippocampal neurons from septic mice (*n* = 3, mean ± SEM, unpaired *t*-test, ***p < 0.001). **E** Fgr was not co-localized with GFAP and Iba1 in the hippocampus of septic mice. Scale bar, 50 μm

To demonstrate the role of Fgr in SAE, the mRNA expression of various Src family kinases was examined in the hippocampus in mice. As shown in Fig. 1A, the mRNA levels of the Src family kinase members Src, Fyn, Yes, Lck, Lyn, and Hck were unchanged in CLP mice, whereas the mRNA levels of the Fgr kinase were significantly increased in these mice. In addition, the expression of Fgr protein was markedly increased in the hippocampus in the CLP mice (Fig. 1B). To verify whether Fgr was expressed in neurons or neuroglia in the hippocampus, double immunofluorescence experiments were performed. Fgr immunoreactivities were consistently very weak or undetectable in sham mice (Fig. 1C). In contrast, sepsis induction by CLP robustly increased the density of Fgr immunoreactivities compared with those of the sham group (Fig. 1C). Most of the Fgr-immunoreactive cells in the hippocampus were co-localized with NeuN (a biomarker for neurons) in CLP mice, but were not co-localized with neuroglia markers (Iba1 for microglia and GFAP for astrocytes) (Fig. 1C–E). This finding indicated that the increased Fgr following sepsis induction was expressed predominantly in hippocampal neurons.

### Pharmacological inhibition of Fgr ameliorates the survival rate and cognitive and emotional dysfunction of CLP-induced septic mice

**Fig. 2:**
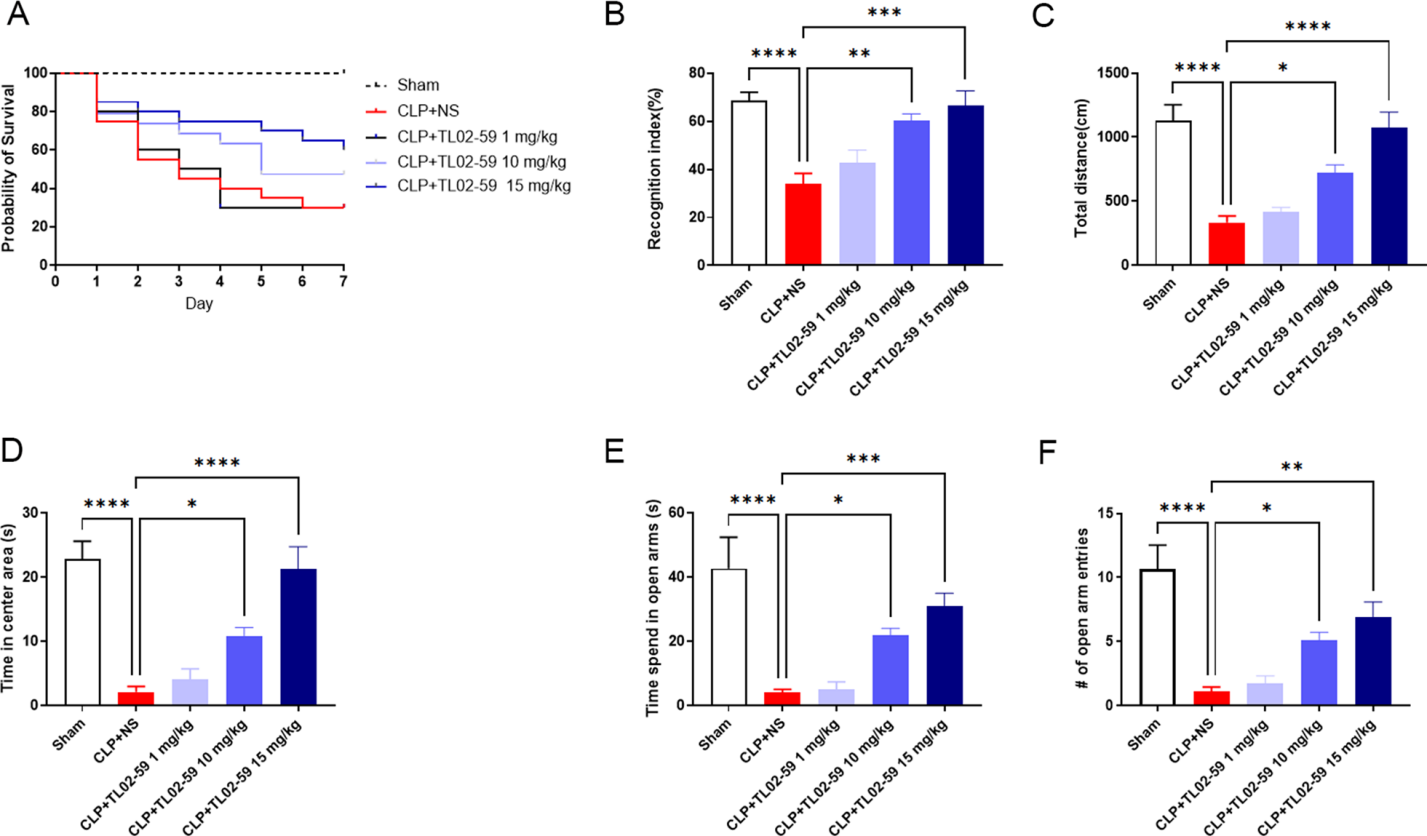
Fgr inhibition ameliorated the survival rate and cognitive and emotional dysfunction of CLP-induced septic mice. **A** The survival rate of sham and septic mice treated with Fgr inhibitor TL02-59 (0 [NS], 1, 10, and 15 mg/kg) was recorded at 7 days. **B**, **C** Total distance and time spent in center areas during 5-min locomotor activity test in the OFT (*n* = 8–10, mean ± SEM, one-way ANOVA with Bonferroni post hoc test, *p < 0.05, ****p < 0.0001). **D** In the NORT, the recognition index of sham and TL02-59 treated septic mice (*n* = 8–10, mean ± SEM, one-way ANOVA with Bonferroni post hoc test, **p < 0.01, ***p < 0.001, ****p < 0.0001). **E**, **F** In the EPM, time spent in open arms and entries of open arms (*n* = 8–10, mean ± SEM, one-way ANOVA with Bonferroni post hoc test, *p < 0.05, **p < 0.01, ***p < 0.001, ****p < 0.0001)

We further examined whether the increased Fgr was associated with the survival rate and cognitive and emotional dysfunction in septic mice. TL02-59 (1, 10, or 15 mg/kg), which is a selective inhibitor of Fgr, was administered through the tail vein 1 h after CLP surgery and once daily for the following 3 days. As expected, the survival rate of the sham group was 100%, whereas the survival rate of the CLP group decreased significantly. However, after TL02-59 treatment, the survival rate markedly improved in comparison with that of the CLP group (Fig. 2A).

Behavioral tests were conducted on the mice 7 days after CLP surgery. First, cognitive function in the mice was assessed by the NORT. The septic mice had a significantly lower recognition index than that of the sham group. In comparison, the recognition index of TL02-59-treated mice was significantly higher than that of septic mice (Fig. 2B). Next, the emotional behaviors of the mice were evaluated using an EPM and OFT. In the OFT, the septic mice exhibited a significantly decreased total distance and time in the central areas compared with the sham group. In contrast, inhibition of Fgr in the sepsis-induced mice markedly increased the total distance and time spent in the central areas compared with those of the CLP group (Fig. 2C, D). In the EPM, septic mice spent significantly less time in open arms and had a lower open arm entry compared with the sham group. However, the duration and entry in open arms were increased following Fgr inhibition when compared with the CLP group (Fig. 2E, F). In addition, these effects were dose dependent. These results suggested that CLP-induced septic mice exhibited cognitive dysfunction and emotional disorders, and inhibition of Fgr prevented these behavioral deficits.

### Fgr inhibitor treatment mitigates microglia activation and inflammatory cytokine responses in septic mice

**Fig. 3:**
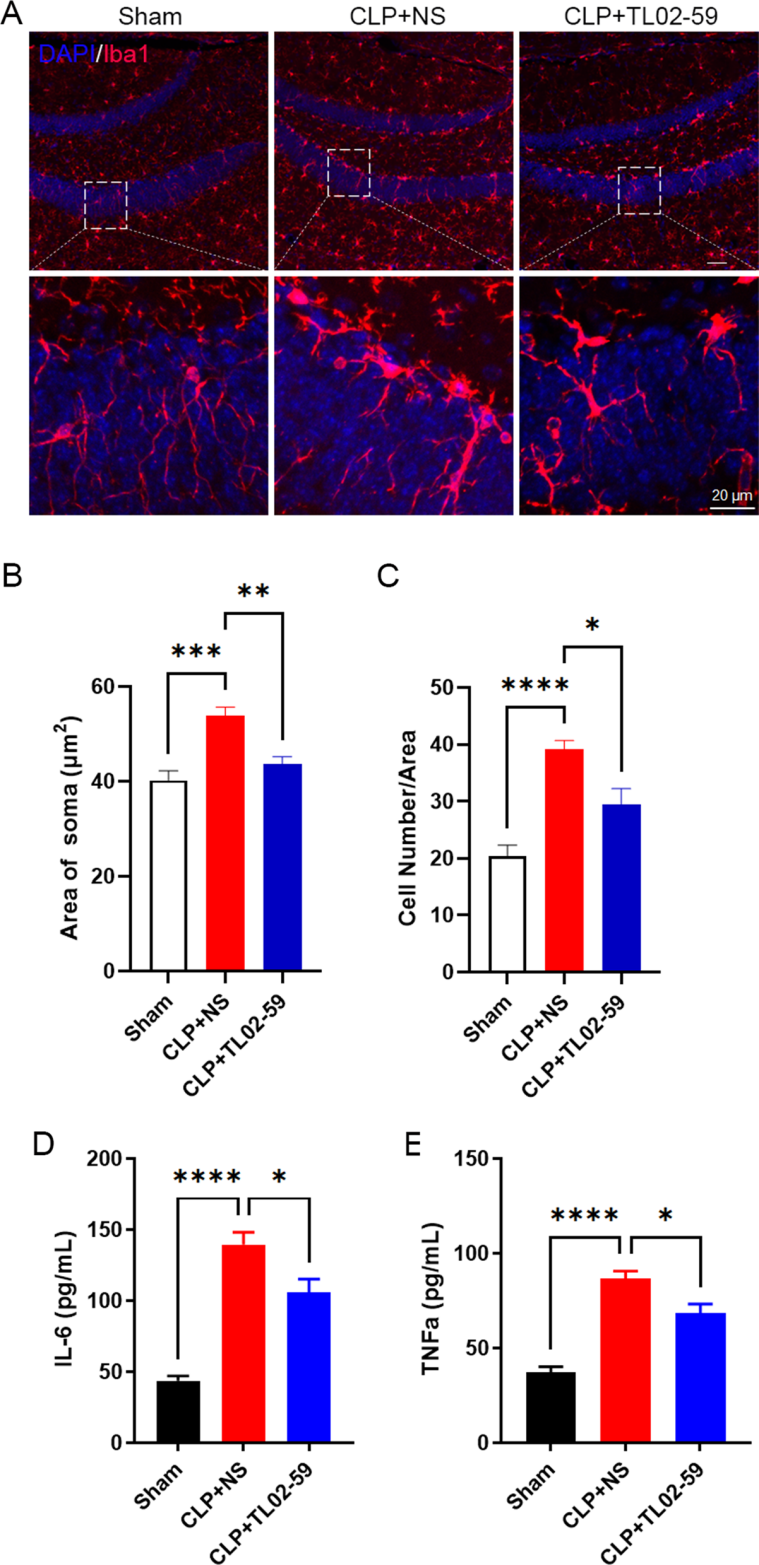
Fgr inhibition diminished microglial activation and neuroinflammation. **A** Representative image of Iba1 (red) and DAPI (blue) fluorescence in the hippocampus at different magnifications (scale bar, 50 μm or 20 μm). **B**, **C** Quantitative analysis of Iba1-positive cell number and soma area (*n* = 5 or 6 mice, mean ± SEM, one-way ANOVA with Bonferroni post hoc test, *p < 0.05, **p < 0.01, ***p < 0.001, ****p < 0.0001). **D**, **E** Levels of IL-6 (**D**) and TNF-α (**E**) in the hippocampus (*n* = 6, mean ± SEM, one-way ANOVA with Bonferroni post hoc test, *p < 0.05, ****p < 0.0001)

Neuroinflammation plays a vital role in the pathology of sepsis [18]. To detect the effect of Fgr on neuroinflammation, immunofluorescent staining was performed to examine microglia activation in the hippocampus. The number and soma area of Iba1-positive microglia were markedly increased compared with those of the sham group, whereas administration of the Fgr inhibitor (15 mg/kg) significantly reduced microglia activation (Fig. 3A–C). In addition, proinflammatory cytokine levels in the brain were investigated using an ELISA to assess the expression of IL-6 and TNF-α in hippocampal tissue. IL-6 and TNF-α expression was elevated in septic mice compared with the sham group, while Fgr inhibitor administration significantly reversed these increases (Fig. 3D, E).

### Inhibition of Fgr suppresses oxidative stress and mitochondrial dysfunction in CLP-induced septic mice

**Fig. 4:**
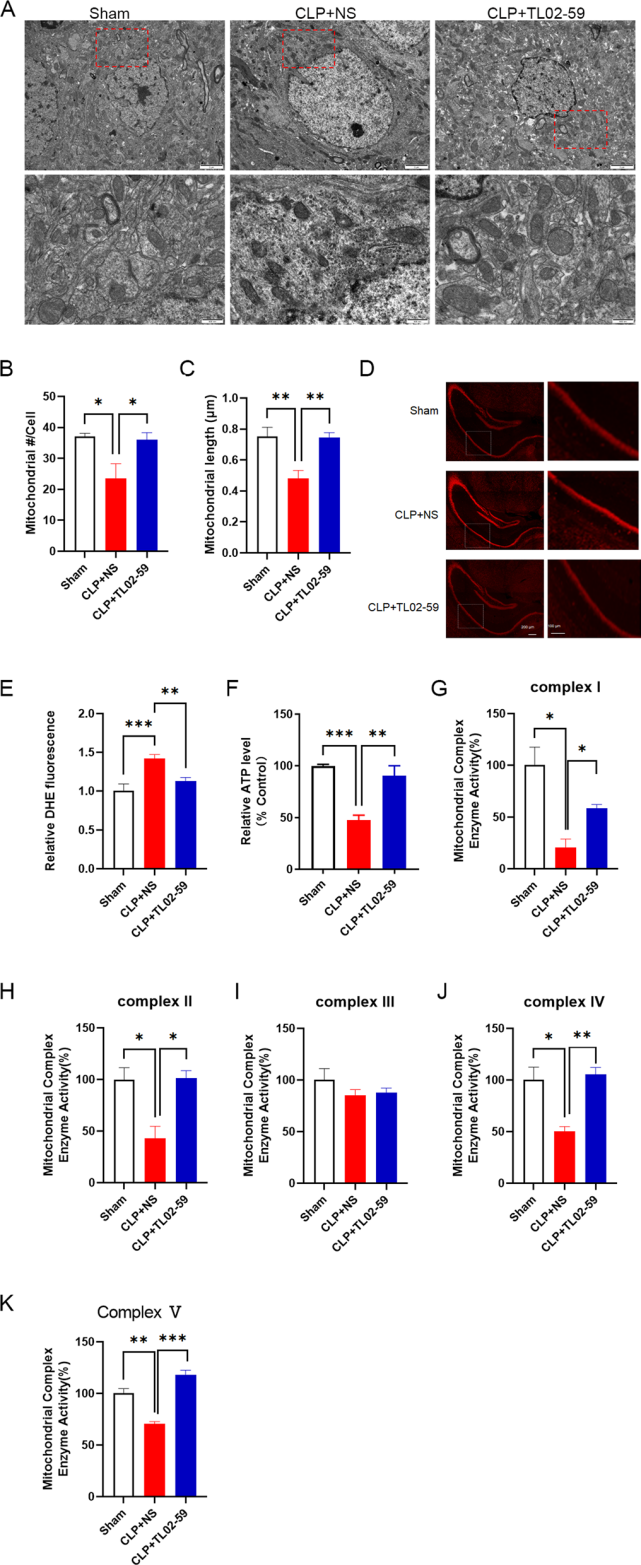
Fgr inhibitor treatment suppressed ROS generation and mitochondrial injury in the hippocampus of septic mice. **A** Representative TEM image of mitochondria in the hippocampus (scale bar, 2 μm or 500 nm). **B**, **C** Statistical analysis of mitochondrial density and length (*n* = 5 mice, mean ± SEM, one-way ANOVA with Bonferroni post hoc test, *p < 0.05, **p < 0.01). **D** Representative DHE staining fluorescence of ROS in the hippocampus (scale bar, 200 μm or 100 μm). **E** Relative level of ROS (normalized to the sham group) (*n* = 5 mice, 3–4 slices per mouse, mean ± SEM, one-way ANOVA with Bonferroni post hoc test, **p < 0.01, ***p < 0.001). **F** ATP content in the hippocampus of the indicated groups (*n* = 5 or 7 mice, mean ± SEM, one-way ANOVA with Bonferroni post hoc test, **p < 0.01, ***p < 0.001). **G**–**K** Mitochondrial respiratory chain complex (I, II, III, IV, and V) enzyme activity in the hippocampus (*n* = 3 mice, mean ± SEM, one-way ANOVA with Bonferroni post hoc test, *p < 0.05, **p < 0.01, ***p < 0.001)

Recent studies showed that activated microglia produced ROS and caused neuron damage [19, 20]. To determine whether the Fgr inhibitor could reduce oxidative stress, DHE staining for ROS was performed in the hippocampus in mice. ROS levels were markedly increased in septic mice compared with the sham group. However, the mice treated with the Fgr inhibitor (15 mg/kg) had a lower level of ROS (Fig. 4D, E). TEM revealed that the number of mitochondria was significantly reduced in the hippocampus of septic mice compared with the sham mice (Fig. 4A, B). The mitochondrial length was also smaller in septic mice (Fig. 4A, C). However, administration of the Fgr inhibitor significantly reversed the damage to the mitochondrial structure (Fig. 4A–C). Next, we investigated whether Fgr was associated with mitochondrial function after CLP surgery. The production of ATP was reduced in septic mice, but markedly elevated in the presence of the Fgr inhibitor (Fig. 4F). In addition, the activity of mitochondrial respiratory chain complexes was detected and revealed that, compared with the sham group, hippocampal mitochondrial complex activity at complex I/II/IV and complex V was significantly reduced in the septic mice (Fig. 4G–K). However, the activity of complex I/II/IV and complex V was significantly reversed after Fgr inhibitor treatment.

### Fgr activates the SIRT1/PGC-1α pathway in CLP-induced septic mice

**Fig. 5:**
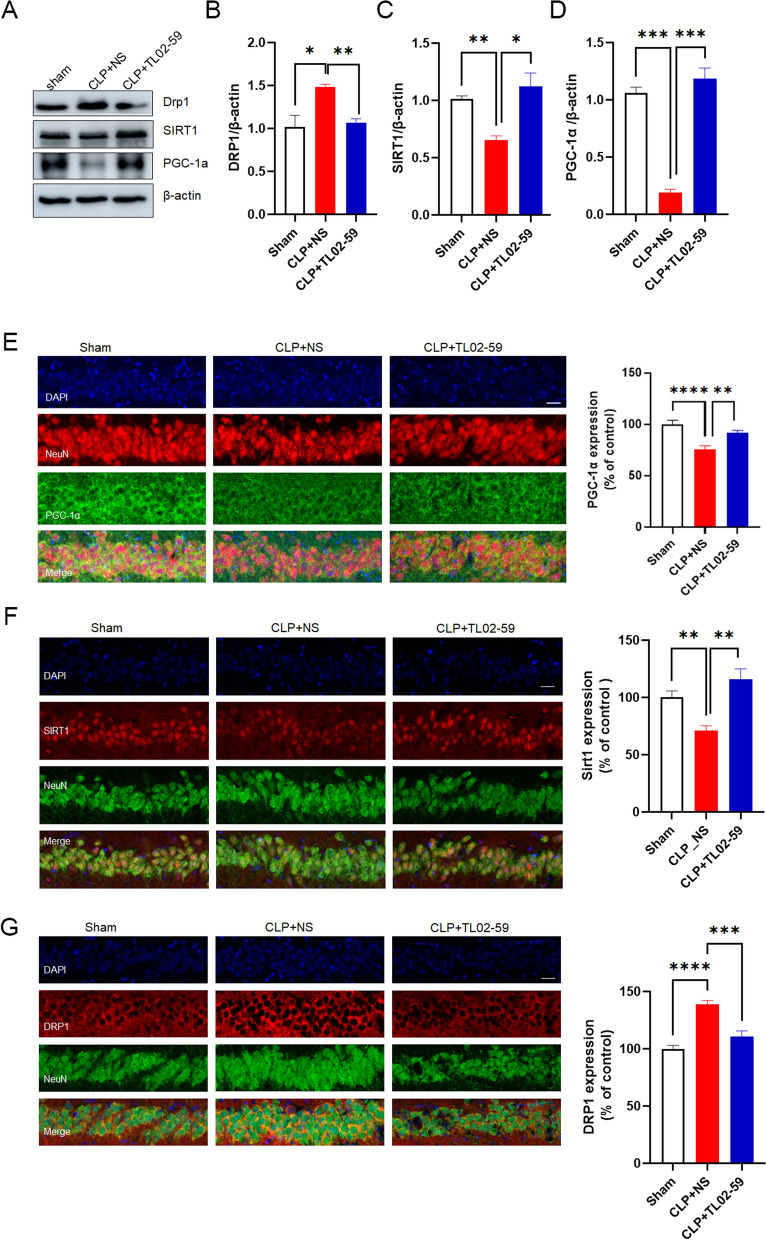
Effects of Fgr inhibitor on hippocampal activation of SIRT1, PGC-1α, and DRP1 pathways in CLP-induced septic mice. **A** Representative Western blot of SIRT1, PGC-1α, and DRP1. **B**–**D** Statistical analysis of SIRT1, PGC-1α, and DRP1 protein levels (*n* = 3 mice, mean ± SEM, one-way ANOVA with Bonferroni post hoc test, *p < 0.05, **p < 0.01, ***p < 0.001). **E**–**G** Representative fluorescent images and quantitative analysis of SIRT1, PGC-1α, and DRP1 in the neuron (labeled with NeuN) (*n* = 5 per group, 3–4 slices per mouse, scale bar = 20 μm, mean ± SEM, one-way ANOVA with Bonferroni post hoc test, **p < 0.01, ***p < 0.001, ****p < 0.0001)

Next, the expression of mitochondrial-related proteins was detected in septic mice. Protein expression of SIRT1 and PGC-1α was reduced in septic mice compared with that in sham mice, but the expression of DRP1 (Dynamin-related protein 1) was increased. Conversely, Fgr inhibitor (15 mg/kg) administration significantly inhibited these alterations in protein expression caused by CLP (Fig. 5A–D). A similar pattern of results was obtained in immunofluorescence staining (Fig. 5E–G).

### Fgr kinase interacts with SIRT1 and regulates its activity

**Fig. 6:**
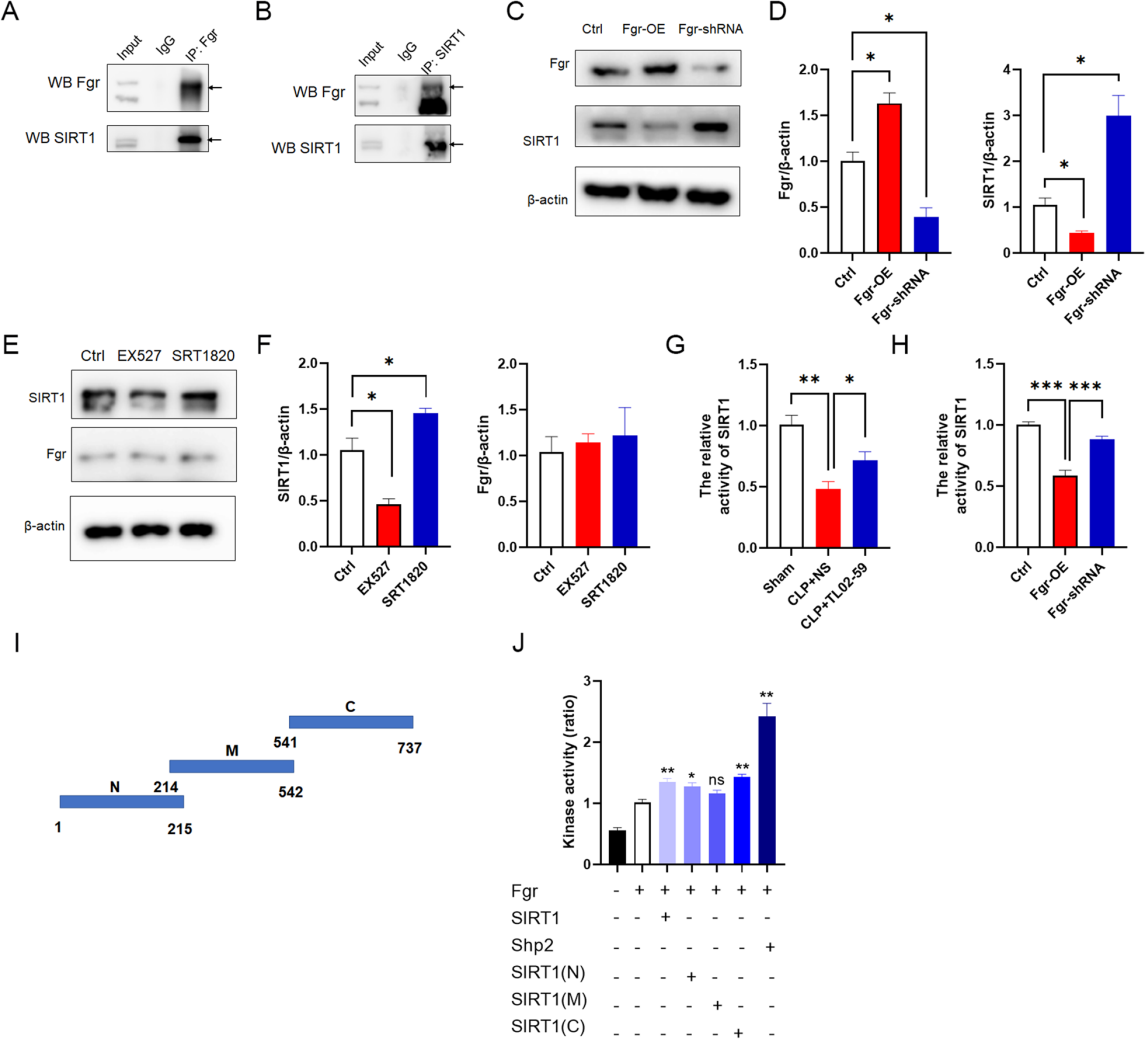
Fgr interacted with SIRT1 and affected its activity. **A**, **B** Co-immunoprecipitation assay indicating that Fgr interacts with SIRT1 in the hippocampus of WT mice in vivo. **C**–**F** Representative Western blot and statistical analysis of Fgr and SIRT1 in HT22 mouse hippocampal neuronal cell line (*n* = 3, mean ± SEM, unpaired *t*-test, *p < 0.05). **G** SIRT1 activity in the hippocampus of septic mice (*n* = 3 or 4 mice, mean ± SEM, one-way ANOVA with Bonferroni post hoc test, *p < 0.05, **p < 0.01). **H** SIRT1 activity in HT22 cells (*n* = 4, mean ± SEM, one-way ANOVA with Bonferroni post hoc test, ***p < 0.001). **I** Schematic representation of mouse SIRT1 fragments. **J** Fgr enzymatic activity measured with a Universal Kinase Activity Kit and Shp2 as a substrate (*n* = 3 or 6, mean ± SEM, one-way ANOVA with Bonferroni post hoc test, *p < 0.05, **p < 0.01)

To explore the relationship between Fgr kinase and SIRT1 in vivo, co-immunoprecipitation experiments were performed using mice hippocampal tissues and showed that Fgr could interact with SIRT1 (Fig. 6A, B). In vitro, overexpression of Fgr reduced SIRT1 expression, and knockdown of Fgr increased SIRT1 protein levels (Fig. 6C, D). Next, we investigated whether SIRT1 affected Fgr expression by measuring Fgr expression after treatment with SIRT1 activator or inhibitor. The expression of Fgr did not change significantly after activation or inhibition of SIRT1 (Fig. 6E, F). Subsequently, SIRT1 activity was measured in vivo and in vitro to explore whether Fgr regulates SIRT1 activity. SIRT1 activity was markedly decreased in the CLP group compared with the sham group but was only dampened in the TL02-59-treated mice, and the difference between the TL02-59-treated mice and the CLP mice was significant (Fig. 6G). In addition, overexpression of Fgr reduced the SIRT1 activity, and knockdown of Fgr increased the SIRT1 activity (Fig. 6H). These findings demonstrated that Fgr regulates SIRT1 expression and activity, but SIRT1 does not affect Fgr. Considering that Fgr, as a kinase, can regulate the activity of SIRT1, we speculated that SIRT1 might be a substrate of Fgr kinase. Consequently, we conducted an kinase activity assay using recombinant mouse Fgr and different truncated mouse SIRT1 proteins to measure the Fgr kinase activity. Shp2, a well-known Src substrate [21], was used as a substrate of Fgr and a positive control. Fgr kinase activity was significantly increased when the N-terminal fragment, the C-terminal fragment, or the full-length mouse SIRT1 were used as substrates (Fig. 6I, J). These results demonstrated that Fgr could phosphorylate mouse SIRT1 and indicated that SIRT1 might be a substrate of Fgr kinase.

### SIRT1 pharmacological activation promotes the protective effect of Fgr inhibition on mitochondrial function

**Fig. 7:**
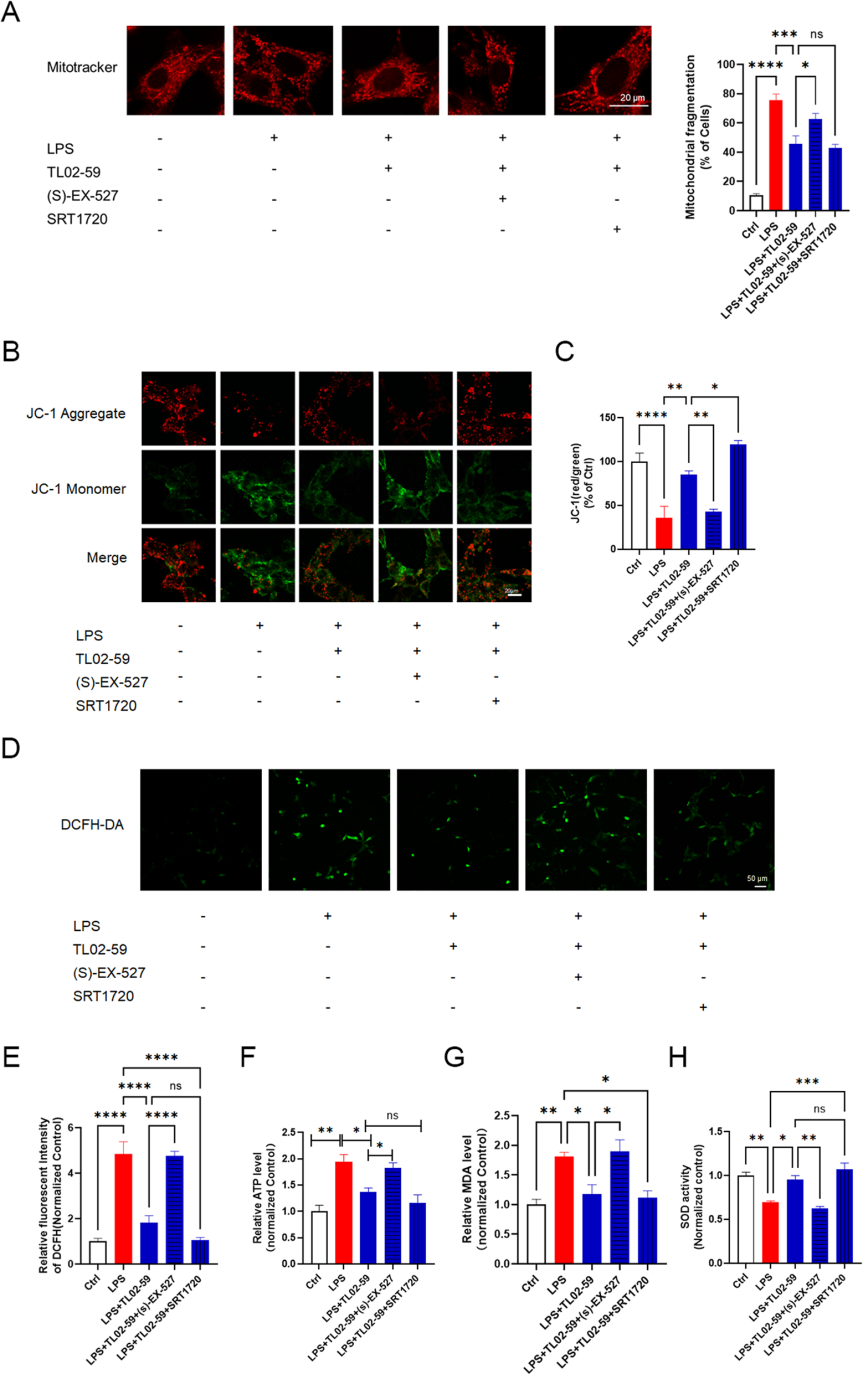
Effects of SIRT1 activation or inhibition on Fgr -mediated protection against oxidative damage and mitochondrial injury. **A** Mitochondrial morphology stained by MitoTracker Deep Red probe (*n* = 5, mean ± SEM, one-way ANOVA with Bonferroni post hoc test, *p < 0.05, ***p < 0.001, ****p < 0.0001). **B**, **C** Mitochondrial membrane potential assayed by JC-1 staining in HT22 cells and statistical analysis (*n* = 5, mean ± SEM, one-way ANOVA with Bonferroni post hoc test, *p < 0.05, **p < 0.01, ****p < 0.0001). **D**, **E** Representative image and statistical analysis of ROS production in HT22 cells stained with DCFH-DA (green) (*n* = 6, mean ± SEM, one-way ANOVA with Bonferroni post hoc test, ****p < 0.0001). **F**–**H** Relative ATP content (**F**), MDA content (**G**), and SOD activity (**H**) in HT22 cells (*n* = 3, mean ± SEM, one-way ANOVA with Bonferroni post hoc test, *p < 0.05, **p < 0.01, ***p < 0.001)

We investigated whether SIRT1 activation was essential for the protective effect of Fgr inhibition on mitochondrial fragmentation by visualizing mitochondrial morphology with the MitoTracker Deep Red probe. Compared with the control group, mitochondrial fragmentation was increased after LPS treatment, and Fgr inhibitor treatment improved this LPS-induced mitochondrial fragmentation (Fig. 7A). In addition, SIRT1 activation/inhibition enhanced or counteracted the protective effects of the Fgr inhibitor, respectively. JC-1 staining showed that the Fgr inhibitor reversed the LPS-induced mitochondrial membrane potential loss, while the SIRT1 inhibitor counteracted the aforementioned variation (Fig. 7B, C). LPS induced considerable oxidative damage—DHE staining and ELISAs showed an increase in the levels of ROS, ATP, and MDA and a decrease in SOD, when compared with the control group (Fig. 7D–H). On the contrary, administration of the Fgr inhibitor reversed these changes, whereas SIRT1 activation promoted the favorable effects of Fgr inhibition on oxidative damage and SIRT1 inhibition counteracted the protective effects of the Fgr inhibitor.

### Pharmacological approaches to modulate PGC-1α regulate the effect of Fgr on mitochondrial injury and cognitive and emotional behaviors

**Fig. 8:**
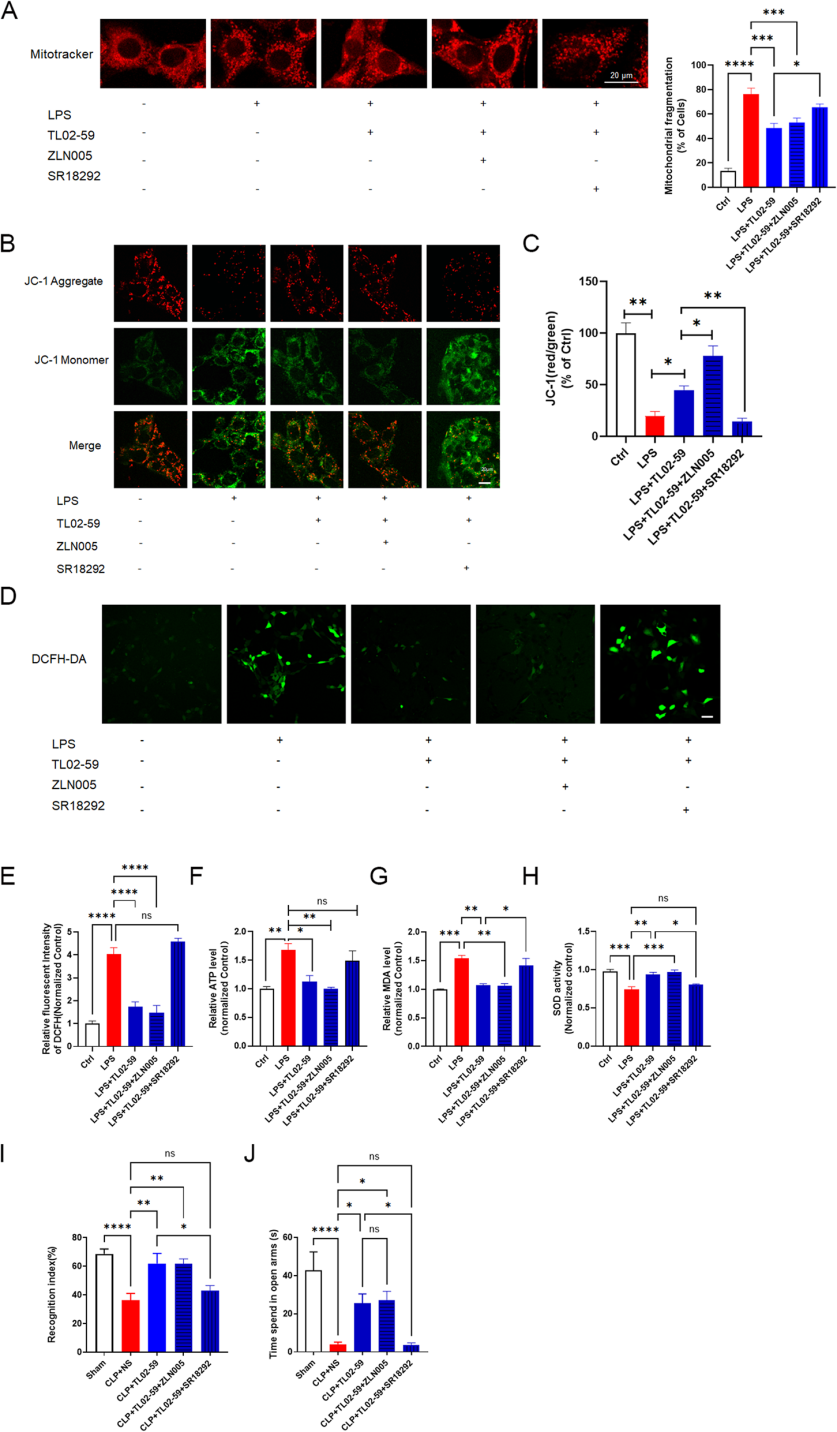
Effects of PGC-1α activation on Fgr-mediated protection of oxidative damage, mitochondrial function, and cognitive and emotional behaviors. **A** Mitochondrial morphology stained by MitoTracker Deep Red probe (*n* = 5, mean ± SEM, one-way ANOVA with Bonferroni post hoc test, *p < 0.05, ***p < 0.001, ****p < 0.0001). **B**, **C** Mitochondrial membrane potential assayed by JC-1 staining in HT22 cells and statistical analysis (*n* = 3, mean ± SEM, one-way ANOVA with Bonferroni post hoc test, *p < 0.05, **p < 0.01). **D**, **E** Representative image and statistical analysis of ROS production in HT22 cells stained with DCFH-DA (green) (*n* = 5–6, mean ± SEM, one-way ANOVA with Bonferroni post hoc test, ****p < 0.0001). **F**–**H** Relative ATP content (**F**), MDA content (**G**), and SOD activity (**H**) in HT22 cells (*n* = 3, mean ± SEM, one-way ANOVA with Bonferroni post hoc test, *p < 0.05, **p < 0.01, ***p < 0.001). **I** Recognition index in different groups (*n* = 7–9 mice, mean ± SEM, one-way ANOVA with Bonferroni post hoc test, *p < 0.05, **p < 0.01, ****p < 0.0001). **J** Time spent in open arms in the EPM (*n* = 8–10 mice, mean ± SEM, one-way ANOVA with Bonferroni post hoc test, *p < 0.05, ****p < 0.0001)

MitoTracker was used to staining live mitochondria. Treatment with the Fgr inhibitor TL02-59 attenuated LPS-induced mitochondrial fragmentation, while administration of the PGC-1α inhibitor SR18292 abolished the effects of the Fgr inhibitor, resulting in reduced mitochondrial fragmentation (Fig. 8A). In addition, the PGC-1α activator ZLN005 promoted the protective effects of the Fgr inhibitor on LPS-induced mitochondrial fragmentation. Next, mitochondrial membrane potential and oxidative stress were investigated in response to PGC-1α modulation. JC-1 staining results showed that the mitochondrial membrane potential of HT22 neurons was decreased in LPS-treated cells. Inhibition of Fgr markedly reversed the decrease in mitochondrial membrane potential induced by LPS treatment. Additionally, the PGC-1α inhibitor or activator significantly counteracted or enhanced, respectively, these effects of the Fgr inhibitor on mitochondrial membrane potential (Fig. 8B, C). Furthermore, the Fgr inhibitor TL02-59 significantly increased the levels of ROS, ATP, and MDA and reduced the level of SOD. Contrarily, administration of the PGC-1α inhibitor or activator counteracted or enhanced these changes, respectively (Fig. 8D–H). Finally, the effects of the PGC-1α inhibitor or activator on Fgr-mediated protection of cognitive and emotional behaviors after CLP surgery were assessed. Compared with CLP + Fgr inhibitor group, the PGC-1α inhibitor counteracted the beneficial effects of Fgr inhibitor administration in NORT and EPM (Fig. 8I–K).

## Discussion

Sepsis-induced brain injury, also known as SAE, has behavioral manifestations ranging from dementia to coma [22]. Understanding the mechanisms underlying this sepsis-induced neurobehavioral impairment may facilitate the development of new treatment strategies for SAE. In recent decades, many studies have been conducted on SAE, but its specific mechanism has not been fully elucidated. Currently, etiological treatment, symptomatic treatment, and supportive therapy are mainly adopted for patients with SAE. The principle is to carry out targeted diagnosis, examination, and treatment to realize early prevention, early detection, and early intervention to reduce the incidence and mortality of sepsis. Branched-chain amino acid infusions [23], plasmapheresis or plasma filtration absorption [24], activated protein C [25], dexmedetomidine [26, 27], and so on, have been tried in human models of SAE, and although these treatments are used in clinical practice, there is still a lack of effective individualized treatment for SAE. In this study, we uncovered a previously unrecognized role for Fgr kinase in SAE. According to our data, pharmacological inhibition of Fgr attenuated CLP-induced neuroinflammation and neurobehavioral dysfunction. In addition, these effects were primarily mediated through SIRT/PGC-1α signaling pathway-mediated oxidative stress and mitochondrial damage induced by Fgr. Hence, our findings prove that Fgr is a neuroprotective agent in SAE. Compared with other experimental therapies, TL02-59 works through multiple pathways, has a half-life of approximately 6 h [15], and is effective at very low concentrations. These factors, coupled with our findings, indicate the strong therapeutic potential of TL02-59 for SAE.

Fgr is a member of the Src family of non-receptor tyrosine kinases. Among these members, Src, Fyn, Yes, Lyn, Lck, and Fgr are expressed in the central nervous system, whereas Blk, Hck, and Lck are more restricted to the tissue [16, 28, 29]. Src kinases are involved in several cellular processes, including proliferation, survival, adhesion, migration, and phagocytosis [30]. Src, Fyn, and Lyn have also been implicated in neuronal development, synaptic plasticity, and signal transduction [31–33]. In addition, Src, Fyn, Lck, Fgr, and Lyn have been associated with neurological disorders, including Alzheimer’s disease, Parkinson’s disease, pain, diabetes, and schizophrenia [29, 30, 33–36]. However, to our knowledge, Fgr has not been reported to contribute to SAE.

CLP-induced sepsis can activate the Fgr gene in the hippocampus. In our study, Fgr expression was low in sham mice, but following CLP surgery, Fgr mRNA and protein levels were markedly increased. Subsequently, we found that this increase predominantly occurred in neurons. However, although CLP surgery appears to activate the Fgr gene, it is unclear how it does so, and the potential mechanism needs to be explored in the future.

CLP surgery was used to induce sepsis in mice in our study. It is well-established that this model causes brain injury and cognitive impairment [37–40]. Consistent with previous studies, our CLP-induced septic mice showed significant cognitive impairment at 7 days in NORT. CLP-induced septic survivors lasted 6 weeks and showed learning and memory impairment [41, 42]. However, some researchers found that CLP mice regained normal memory ability 45 days after the operation [42, 43]. This suggests that some survivors may recover from brain damage to a certain extent over time. In previous studies, septic mice developed anxiety-like behavior, but this was resolved at 45 days post-sepsis [44]. In this study, inhibition of Fgr ameliorated the cognitive dysfunction and anxiety-like behavior at 7 days, indicating that brain function was recovered. Neuroinflammation is one of the most critical central mechanisms in SAE, in which the activation of microglia is instrumental [45, 46]. Activated microglia release proinflammatory cytokines, which further aggravate brain dysfunction. Therefore, inhibiting the activation of microglia and neuroinflammation may be an effective strategy for treating SAE. Our results showed that CLP could induce microglia activation, and that inhibition of Fgr markedly reduced this microglia activation and the release of proinflammatory cytokines.

ROS are essential in the process of inflammatory disorders, including neurodegeneration and cancer [47]. Abnormal ROS signal transduction or excessive ROS production can affect the pathophysiology of a disease [48]. Previous studies showed that ROS enhancement is the primary cause of tissue injury in sepsis [49, 50]. Therefore, we explored whether inhibition of Fgr could reduce neuroinflammation by inhibiting the oxidative stress caused by ROS production. We found that ROS levels were increased after CLP surgery, while Fgr inhibitor administration markedly reduced ROS production. Mitochondria are the main source of ROS production, and mitochondrial ROS are involved in redox signal transduction [51, 52]. Mitochondrial dysfunction is related to the excessive increase of ROS, which further aggravates mitochondrial dysfunction and neuroinflammation, and finally triggers various diseases [53, 54]. Our TEM results showed that the number and length of mitochondria were significantly reduced in septic mice, implying that mitochondrial damage may contribute to increased ROS. In addition, inhibition of Fgr markedly rescued mitochondrial damage and decreased ROS production in the hippocampus. Mitochondrial respiratory chain complexes are the main source of ROS. The activity of these complexes affects ROS production and mitochondrial function, which leads to the loss of membrane potential, disturbances in oxidative phosphorylation, and a decrease in ATP production [55–57]. Our data showed that CLP surgery decreased ATP production and mitochondria complex I/II/IV/V activity, while Fgr inhibitor treatment reversed the decrease in vivo. Thus, our results suggest that antagonism of CLP-induced neuroinflammation after intervention with Fgr may improve mitochondrial function and reduce ROS production.

SIRT1, a member of the sirtuin family, is an NAD^+^-dependent protein deacetylase that is involved in different biological processes, including inflammation, mitochondrial biogenesis, energy management, aging, and cell death [58, 59]. SIRT1 deacetylates downstream PGC-1α to increase its activity [60, 61]. The SIRT1/PGC-1α interaction is important in oxidative stress, mitochondrial biogenesis and function, and anti-aging processes [60, 62, 63]. Previous studies showed that SIRT1 and PGC-1α expression was reduced in the peripheral tissues of septic mice [64–67]. Consistent with such studies, our results showed that SIRT1/PGC-1α protein levels were decreased in the hippocampus of septic mice, and inhibition of Fgr could reverse this decrease. To further explore the mechanism of Fgr regulation of SIRT1, we performed a co-immunoprecipitation to examine the relationship between Fgr and SIRT1. In addition, further kinase activity analysis revealed that Fgr regulated SIRT1 activity and suggested that SIRT1 might be the substrate of Fgr kinase.

The neuroprotective effect of SIRT1 and PGC-1α in acute and chronic neurodegenerative diseases has been studied in detail [68, 69]. SIRT1 overexpression or activation was shown to protect against nervous system injury, including cerebral ischemia and traumatic brain injury, by deacetylating its target proteins, including FOXO1, PGC-1α, p53, NF-κB, AMPK, and VEGF [70–73]. In the brain, SIRT1 is highly expressed in the hippocampus, hypothalamus, cortex, and cerebellum [73]. In this study, SIRT1 was expressed in hippocampal neurons, and its expression markedly increased after CLP surgery. Furthermore, SIRT1 activation markedly enhanced the neuroprotective effect of the Fgr inhibitor on mitochondrial structure and function. However, SIRT1 inhibition counteracted the protection by the Fgr inhibitor. Thus, SIRT1 activation might be beneficial against injury in sepsis. On the contrary, some studies have shown that SIRT1 may play an adverse role in sepsis. Liu et al. reported that SIRT1 expression was increased in human monocytes and leukocytes from patients with sepsis, and decreased SIRT1 protein levels enhanced the inflammatory response [74]. Other studies demonstrated that SIRT1 inhibition could restore repressed endothelial E-selectin, ICAM-1, and PSGL-1 expression on neutrophils, reduce cytokine production, and enhance survival rate of septic mice [75–77]. The variation in the observed effects of SIRT1 in sepsis may be due to the different treatment times of SIRT1; that is, SIRT1 needs to be treated according to the different stages of the disease. PGC-1α, a downstream target of SIRT1, is a transcription factor that regulates mitochondrial function and protects neurons from oxidative stress [78]. In our study, PGC-1α activation promoted the protective effect of Fgr on mitochondrial injury and cognitive and emotional behaviors. In addition, PGC-1α inhibition counteracted the above-mentioned phenomenal. Thus, the treatment effects of the Fgr inhibitor on SAE might be mediated by activation of the SIRT1/PGC-1α signaling pathway.

There are several limitations in our study. First, all the experiments were conducted in mice, and the applicability of the findings needs to be further explored in patients with sepsis. Second, considering the large individual differences in the time window of the inflammatory response in septic patients and the different clinical manifestations in various stages of the disease, the activation treatment of SIRT1 or PGC-1α should be based on the characteristics of the immune function of patients to determine a reasonable time of action. Third, only CLP was used to establish an SAE model in this study, and the activation of the Fgr/SIRT1/PGC-1α pathway may play a different role in sepsis induced by other factors.

## Conclusions

In summary, our results suggested that Fgr kinase inhibition markedly ameliorated the survival rate, cognitive and emotional dysfunction, neuroinflammation, oxidative stress, and mitochondrial damage through activation of the SIRT1/PGC-1α pathway. These findings imply that Fgr is a promising therapeutic target for SAE.

## Data Availability

All data generated or analyzed during this study are included in this published article.

## Abbreviations

CLP: Cecal ligation and puncture
DHE: Dihydroethidium
EPM: Elevated plus maze
NORT: Novel object recognition test
OFT: Open field test
SAE: Sepsis-associated encephalopathy
TEM: Transmission electron microscopy
